# Supplementary material for: Enhanced superconductivity and coexisting ferroelectricity at oxide interfaces
Source: Nat Commun. 2025 Dec 1;17:219. doi: 10.1038/s41467-025-66903-5 (PMC12779636; doi:10.1038/s41467-025-66903-5)
Supplement: Supplementary file 1 — Supplementary Information [file 41467_2025_66903_MOESM1_ESM.pdf]

Supplementary Information for

Enhanced superconductivity and coexisting ferroelectricity at oxide interfaces

Meng Zhang *et al.*

*Corresponding authors: physmzhang@zju.edu.cn; ywxie@zju.edu.cn

This file includes:

Supplementary Note 1

Figs. S1 to S6

Supplementary Note 1: Raman scattering measurements in LAO/KTO heterostructures

(i) KTO phonon modes

Three transverse polar optical phonon modes (TO_1, TO_2, and TO_4) are theoretically predicted and experimentally observed in perovskite KTO systems, including single crystals[1] and ceramics[2]. The TO_1 mode corresponds to the relative vibration of Ta^{5+} ions against rigid O_6 octahedra (Slater mode), TO_2 reflects K^+ ion motion against a rigid TaO_6 framework (Last mode), and TO_4 involves the bending of O_6 octahedra (Axe mode)[2–4]. While the soft TO_1 mode strongly softens upon cooling and exhibits low intensity, TO_2 and TO_4 show stable frequencies but gradually diminishing intensities with increasing temperature. Notably, these *Raman-inactive* modes in ideal KTO are consistently observed and attributed to polar nanoregions (PNRs)[1,2,4–6].

(ii) Experimental results

To directly demonstrate the existence of PNRs in our LAO/KTO heterostructures, we conducted Raman spectroscopy measurements—a well-established technique for detecting PNRs in KTO. For comprehensive comparison, we examined (i) as-grown LAO/KTO, (ii) poled LAO/KTO, (iii) KTO single-crystalline substrate, and (iv) commercial ferroelectric $KTa_{0.65}Nb_{0.35}O_3$ ($KTN_{0.35}$) single crystal. Electrostatic poling was performed at -180 V and 4.5 K in a vacuum cryostat. Following poling, the sample was rapidly warmed to room temperature for Raman measurements to minimize relaxation effects (Fig. S3 confirms that brief ambient exposure does not fully erase the poled state). All spectroscopic data are presented in Fig. S4 with detailed analysis in subsequent subsections.

As shown in Fig. S4a, the commercially available ferroelectric $KTN_{0.35}$ (111) single crystals exhibit prominent TO_2 and TO_4 modes, indicative of symmetry breaking. These modes persist up to ~ 175 K, confirming the ferroelectric character. In the KTO(111) substrates, weaker TO_2 (~ 198 cm^{-1}) and TO_4 (~ 544 cm^{-1}) peaks are observed until ~40 K (Fig. S4b), consistent with previous studies attributing them to PNRs. Crucially, the

LAO/KTO(111) heterostructures exhibit similar features (Fig. S4c), with the TO_4 peak persisting to ~ 60 K post-poling (Fig. S4d).

To enhance the visibility of these features, we performed background subtraction (using high-temperature references) and data normalization. The processed TO_4 peak intensities versus temperature are shown in Fig. S4e. At 60 K, the poled sample shows a distinguishable TO_4 peak (Fig. S4f), absent in the unpoled case; at 1.6 K, the TO_4 peak is stronger in the poled sample than the unpoled one (Fig. S4g). The enhanced intensity and elevated T^*(temperature where transverse polar optical phonon modes emerge) demonstrate improved PNR alignment after poling.

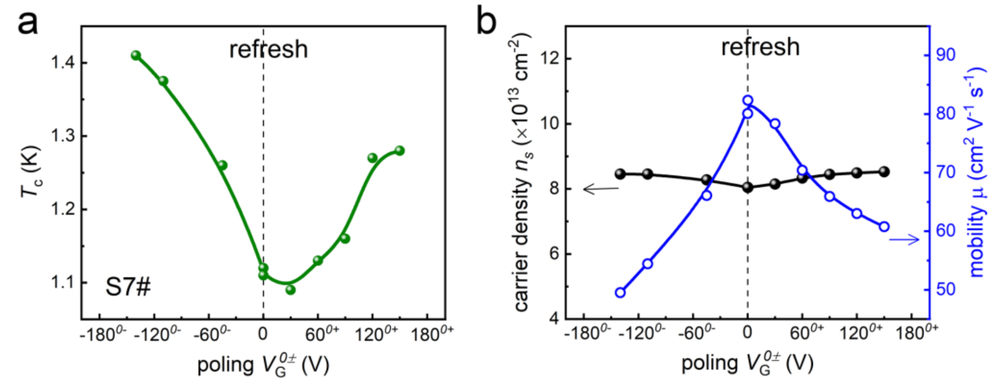

Fig. S1 | Poling a LAO/KTO(111) sample (S7#) with increasing $|V_G|$ from 0 to 180 V for both polarities. The "origin" state was restored through a "refresh" process by leaving the sample in ambient conditions for several days. **a,** Evolution of T_c as a function of poling V_G. **b,** Evolutions of carrier density (n_s, black closed circles) and mobility (μ, blue open circles) as functions of poling V_G.

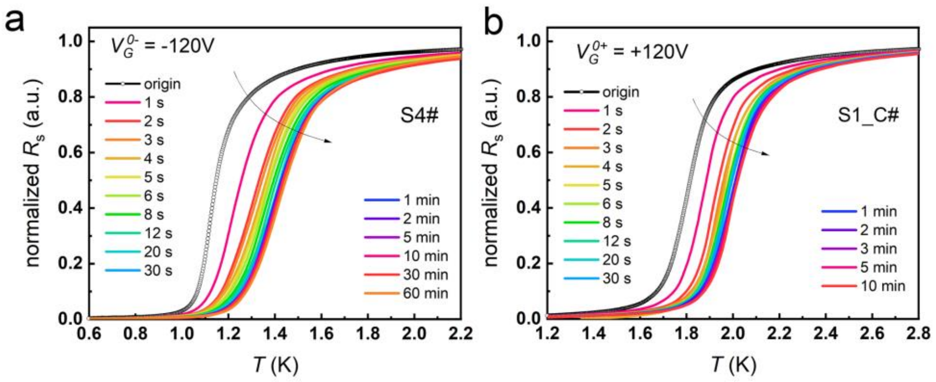

Fig. S2 | Evolution of normalized $R_s(T)$ curves with increasing cumulative poling time. The poling processes were conducted at T = 4.5 K, with cumulative poling times as labeled. **a,** Results for a poling V_G of -120 V. **b,** Results for a poling V_G of +120 V.

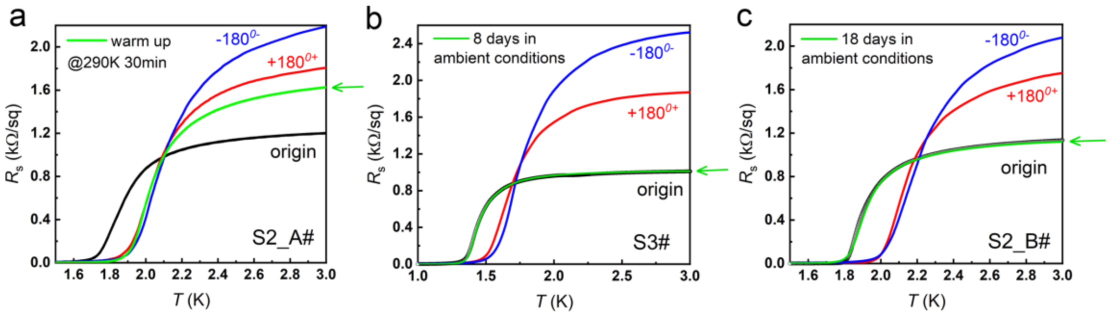

Fig. S3 | Recovery behavior of the poling-induced state. a, Temperature-dependent $R_s(T)$ curve after warming the sample to 290 K and holding it for 30 minutes in the cryostat chamber; **b,** $R_s(T)$ curve after leaving the sample in ambient conditions for 8 days; **c,** $R_s(T)$ curve after leaving the sample in ambient conditions for 18 days. For each sample, the temperature-dependent $R_s(T)$ curves corresponding to "origin", "$+180^{0+}$" and "-180^{0-}" states are shown for comparison.

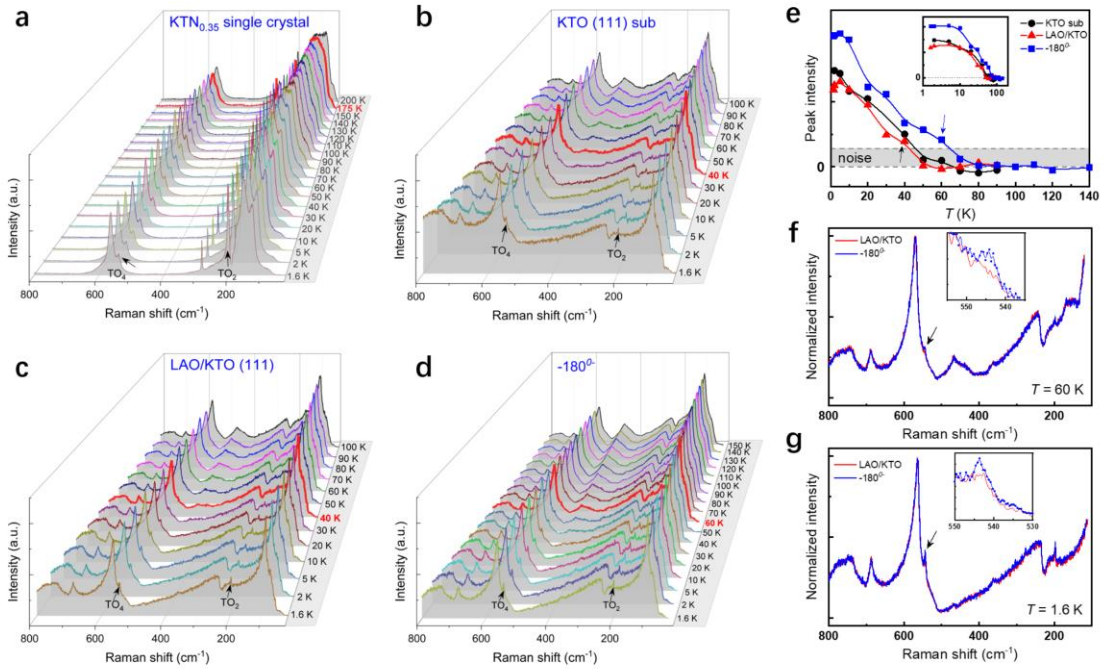

Fig. S4 | Raman spectroscopy. Temperature dependence of normalized Raman spectra in ferroelectric $KTa_{0.65}Nb_{0.35}O_3$ ($KTN_{0.35}$) (**a**) and nominally cubic KTO single crystal substrates (**b**), along with the original (**c**) and 0^- state (poling voltage -180 V) (**d**) of the LAO/KTO (111) heterostructure. Bold red curves are highlighted for clarity, indicating the temperature (T^*) where the transverse polar optic phonon modes, TO_2 and TO_4, appear. The frequencies of the polar phonon modes are marked by arrows. **e,** TO_4 peak intensity as a function of temperature, obtained by background subtraction from high-temperature spectra. The inset shows that the peak intensity tends to saturate below 10 K. Comparison of the original and 0^- state spectra at temperatures of 60 K (**f**) and 1.6 K (**g**), with zoomed-in view of the TO_4 vicinity shown in the insets.

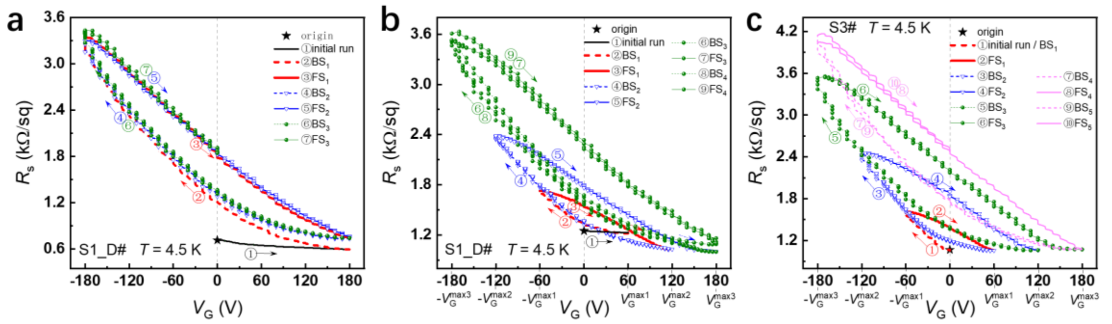

Fig. S5 | Ferroelectric-like hysteresis independent of first sweep polarity in back-gated LAO/KTO. a-c, V_G-R_s characteristics of LAO/KTO heterostructures exhibit stable bistable states (**a**) and a systematic increase in hysteresis width with increasing maximum gate voltage (V_G^{max} = 60, 120, 180 V), irrespective of the polarity of the initial voltage sweep (**b, c**). The hysteresis loops ultimately stabilize into a reproducible double-state configuration at zero bias ($0^{\pm}$), consistent with robust ferroelectric polarization switching behavior. FS: forward sweep; BS: backward sweep.

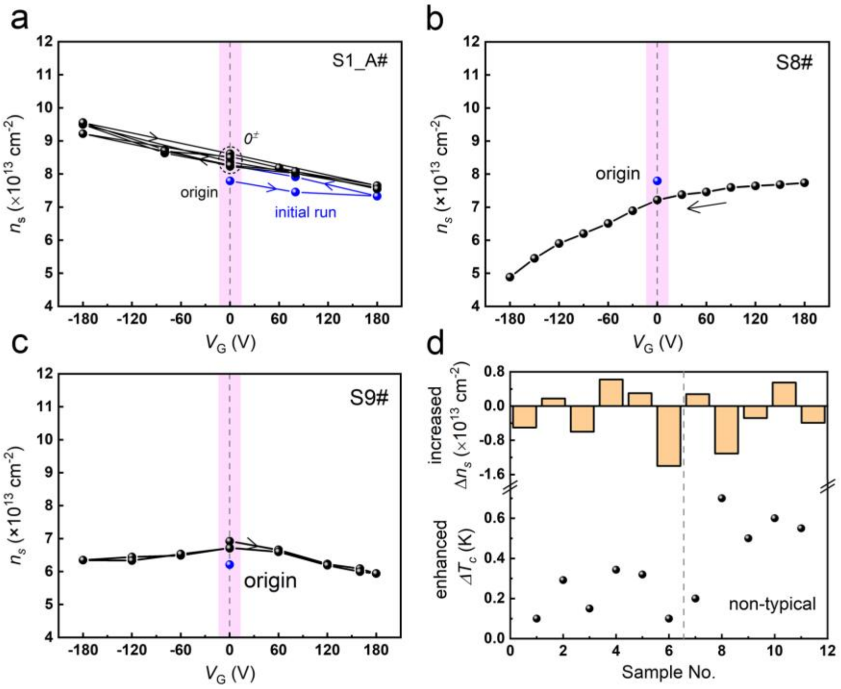

Fig. S6 | Evolution of carrier density (n_s) with gating V_G in various LAO/KTO samples. The pink shading highlights the "$V_G = 0$" region. **a,** Sample S1_A# during the continuous gating cycles described in the main text and Fig. 2. **b,** Sample S8#. **c,** Sample S9#. **d,** Summary of Δn_s, the difference in n_s between the "$0^{\pm}$" states and their corresponding "origin" state, across various samples. Two key points are noteworthy: (1) As illustrated in panels **a-c**, the n_s-V_G relationship varies in different samples, supporting the conclusion that the primary effect of V_G is not on n_s through a simple capacitance mechanism. Instead, n_s is influenced by several factors, the detailed discussion of which is beyond the scope of this study. (2) As shown in panel **d**, while the enhancement in T_c (indicating the presence of ferroelectric polarization) is a universal feature, Δn_s can be either positive or negative. This strongly supports the conclusion that the observed phenomena are not driven by changes in n_s. The less change in n_s of 0^+ further rules out charge trapping mechanism, as removal of a positive V_G in typical scenarios leads to carrier depletion and a significant reduction in concentration[7,8]. The term "non-typical" is used as defined in **Fig. 1b**.

References

1. Uwe, H., Lyons, K. B., Carter, H. L. & Fleury, P. A. Ferroelectric microregions and Raman scattering in $KTaO_3$. *Phys. Rev. B* **33**, 6436–6440 (1986).
2. Glinšek, S. *et al.* Lattice dynamics and broad-band dielectric properties of the $KTaO_3$ ceramics. *J. Appl. Phys.* **111**, 104101 (2012).
3. Nilsen, W. G. & Skinner, J. G. Raman spectrum of potassium tantalate. *J. Chem. Phys.* **47**, 1413–1418 (1967).
4. Wen, X. *et al.* Unveiling the coupling effect of strain behavior and electro-optic property in nanodisordered KTN crystal. *Appl. Phys. Lett.* **122**, 232905 (2023).
5. Kojima, S., Rahaman, Md. M., Sase, R., Hoshina, T. & Tsurumi, T. Vibrational dynamics of ferroelectric $K(Ta_{1-x}Nb_x)O_3$ studied by far-infrared spectroscopic ellipsometry and Raman scattering. *Jpn. J. Appl. Phys.* **57**, 11UB05 (2018).
6. Aktas, O., Crossley, S., Carpenter, M. A. & Salje, E. K. H. Polar correlations and defect-induced ferroelectricity in cryogenic $KTaO_3$. *Phys. Rev. B* **90**, 165309 (2014).
7. Biscaras, J. *et al.* Limit of the electrostatic doping in two-dimensional electron gases of $LaXO_3$(X = Al, Ti)/$SrTiO_3$. *Sci. Rep.* **4**, 6788 (2014).
8. Yin, C. *et al.* Electron trapping mechanism in $LaAlO_3$/$SrTiO_3$ heterostructures. *Phys. Rev. Lett.* **124**, 017702 (2020).
